# Acoustic and perceptual evidence relevant to sexual selection in human voice dimorphism

**DOI:** 10.3389/fpsyg.2026.1938866

**Published:** 2026-09-30

**Authors:** Chun He, Qiyuan Tang, Wenting Xian

**Affiliations:** School of Liberal Arts, Guangxi University, Nanning, China

**Keywords:** formant dispersion (Df), fundamental frequency (F0), honest signal, sexual dimorphism, sexual selection

## Abstract

Male and female voices differ markedly in pitch and timbre. Previous work suggests that fundamental frequency (F0) and formant dispersion (Df) correlate with body size, but whether these associations hold within each sex and whether subtle acoustic changes affect social perception remain unclear. We integrate acoustic measurements and perceptual experiments to dissect the roles of F0 and Df in signaling height and guiding social judgments. Acoustic data from 40 males and 40 females showed that F0 exhibited a substantial sex difference but little evidence of a within-sex association with height, whereas Df showed substantial sex differences and more limited, vowel-specific within-sex height associations, with the clearest evidence observed for female /a/ Df. In the perception module, 90 raters evaluated voices manipulated by ±1 just-noticeable difference (JND) in F0 or Df. Across the four evaluator–stimulus conditions, Df produced larger estimated perceptual effects than F0 within each corresponding rating task. Together, these findings suggest that Df may retain some height-related information while contributing to social voice perception.

## 1 Introduction

Adult male and female voices differ markedly in fundamental frequency (F0) and formant structure, a pattern often referred to as vocal sexual dimorphism^1^ (de Campos et al., 2013). Typically, males have lower F0 and lower, more closely spaced formants than females, reflecting well-established sex differences in vocal acoustics and sex-related differences in vocal fold and vocal tract morphology that become pronounced during puberty (Fant, 1960; Fitch and Giedd, 1999; Kent et al., 2023; Suire et al., 2021; Laver, 2012; Marchal, 2009). These developmental changes are strongly modulated by sex hormones, most notably androgen dependent laryngeal growth and vocal fold restructuring in males (Barbier et al., 2015; Styne et al., 1998; Hirano et al., 1981). Because vocal tract dimensions scale with body size, vocal sexual dimorphism has often been partly attributed to anatomical scaling (Fitch, 1997; Fitch and Giedd, 1999).

Yet human vocal dimorphism appears larger than what simple height scaling would predict. Rendall et al. (2005) showed that the female-to-male F0 ratio is about 1.81, substantially higher than the corresponding height ratio of roughly 1.09, and found little evidence that F0 covaries with height within each sex. This suggests that the magnitude of human F0 dimorphism cannot be explained solely by proportional body-size scaling. Comparative work further indicates that the unusually large sex difference in human F0 differs from the more proportional acoustic allometry seen in non-human primates (Fitch, 1997; Puts et al., 2016; Owren and Rendall, 2001; Houde and Endler, 1990). Thus, while F0 offers a useful benchmark for studying human vocal sexual dimorphism, whether other acoustic dimensions retain within-sex information about height remains less clear.

One relevant dimension is formant dispersion (Df), which reflects the overall spacing of vocal tract resonances and has been linked to vocal-tract dimensions and body size in humans and other primates (Puts et al., 2011a; Puts et al., 2011b). Unlike F0, which is heavily influenced by vocal fold length, mass, and tension, Df is more directly tied to vocal tract configuration (Fant, 1960; Fitch and Giedd, 1999). However, evidence for its relationship to body size in humans is less consistent and less thoroughly examined than for F0. Critically, although Rendall et al. (2005) established that human F0 dimorphism exceeds height dimorphism and shows little within-sex height correlation, they did not systematically test Df across sexes or vowels. Whether Df preserves height-related information within sex therefore remains a distinct empirical question, and is the primary acoustic issue we address here.

The evolutionary significance of vocal sexual dimorphism also hinges on whether acoustic differences are perceptually detectable and behaviorally relevant (McAleer and Belin, 2019; Semple and McComb, 2000; Bestelmeyer, 2019; Ghazanfar and Hauser, 2001; Michalsky and Schoormann, 2021). Sexual dimorphism in primates is often linked to sexual selection, particularly through male–male competition (Plavcan, 2012). In humans, lower F0 and lower Df can enhance perceived dominance and aggressiveness, and both parameters have been associated with upper-body strength and testosterone-related markers of competitive ability (Puts et al., 2006, 2007; Jones et al., 2010). Female vocal characteristics also shape social evaluations: higher-pitched, more feminine voices tend to be rated as more attractive by males (Apicella and Feinberg, 2009; Feinberg et al., 2008; Šturm et al., 2026; Collins and Missing, 2003), and female vocal traits can affect judgements relevant to attractiveness and intrasexual competition (Puts et al., 2011a; Puts et al., 2011b; Barkat-Defradas et al., 2021; Shirazi et al., 2018; Barron-Lutzross, 2018). These findings indicate that both F0 and Df can influence socially relevant judgements, but they do not by themselves establish that either feature evolved via sexual selection. Because sexually dimorphic traits may also arise as developmental by-products of hormonal action (Slocombe and Zuberbühler, 2007), evidence that listeners systematically respond to acoustic variation is a useful but incomplete component of an evolutionary account.

In the present study, we therefore ask how F0 and Df compare across acoustic production and social perception, focusing on whether the two parameters convey different information about height and social judgements. We used three complementary modules. Module 1 examined sex differences in F0 and Df and tested their within-sex associations with height. This analysis both replicates the established F0 pattern reported by Rendall et al. (2005) and, more critically, provides a systematic test of Df as a potential height-linked acoustic dimension. Module 2 quantified listeners’ perceptual sensitivity to changes in F0 and Df, providing empirically grounded stimulus magnitudes for the subsequent experiment. Module 3 tested whether controlled shifts in these parameters influence judgements of attractiveness, aggressiveness, and flirtatiousness.

Specifically, we addressed three questions:

*Q1*: Do sex differences in F0 and Df exceed what would be expected from the observed sex difference in height, and is either parameter associated with height within sex?

*Q2*: Are changes in F0 and Df perceptually discriminable at the magnitudes we experimentally manipulate?

*Q3*: Do shifts in these acoustic dimensions towards more typically male or female ranges affect socially relevant judgements of attractiveness, aggressiveness, and flirtatiousness?

Together, these analyses allow us to distinguish the well-established sex difference in F0 from the less understood role of Df, and to evaluate whether the two acoustic dimensions contribute differently to vocal social perception in humans.

## 2 Method

Terminological note. Throughout this paper, we use “sex” to refer to the binary sex categories reported by participants at recruitment (male/female). Moreover, we use “gender” to refer to gender identity or expression, which was not independently assessed in this study. Accordingly, the findings should be interpreted within the sampled sex categories rather than generalized to gender identity or expression. We acknowledge that voice perception and production are also influenced by gender identity and expression, which are distinct sociocultural constructs. Our participant recruitment and screening relied on self-reported biological sex, and our findings should be interpreted within this binary framework.

Following previous work (Puts et al., 2011a; Puts et al., 2011b; Feinberg et al., 2005), we use “masculinized” voices to refer to acoustic manipulations that lower F0 and/or formant dispersion (Df), moving them toward the typical male range, and “feminized” voices to refer to manipulations that raise F0 and/or Df, moving them toward the typical female range (Puts et al., 2011a; Puts et al., 2011b).

Because the present study measured height but not other anthropometric dimensions, we use “height” when referring to the observed variable and “body size” only when discussing the broader construct for which height served as a proxy.

The experiment consisted of three modules. Module 1 aimed to obtain high-fidelity acoustic parameters of natural voices and to address Question 1. Module 2 measured the just-noticeable difference (JND) for changes in F0 and Df. Module 3 used the original recordings collected in Module 1, which were standardized and manipulated, and then employed as experimental stimuli in behavioral experiments designed to investigate questions regarding voice perception in intersexual and intrasexual contexts, respectively.

Participants in the three modules were independent samples; each individual took part in only one module. All participants were naïve to the purpose of the experiment and had no prior experience with similar tasks. Written informed consent was obtained from all participants prior to the experiment, and participation was voluntary. The study protocol was approved by the Institutional Review Board of Guangxi University (Approval No. 2026–060). All participants provided written informed consent and received monetary compensation approved by the IRB: ¥30 per module for each participant.

## 3 Module 1: testing the asymmetry between male/female voices and height

### 3.1 Participants

A total of 82 adult participants were recruited. Two participants were excluded because their median fundamental frequency could not be reliably extracted using the Praat autocorrelation method, resulting in a final sample of 80 individuals (40 females, 40 males). The mean age was 20.43 years for females and 20.75 years for males (Cohen’s *d* = 0.126, indicating a negligible age difference between groups). None reported any history of speech or hearing disorders. Height was recorded for each participant. To control for potential hormonal influences on voice production, all female speakers were recorded during the ovulatory phase of their menstrual cycle (days 13–15 of a self-reported 28-day cycle). This phase was chosen because it corresponds to peak estrogen levels that have been associated with optimal vocal fold hydration and stability (Raj et al., 2010). Although hormonal contraceptive use was not assessed at the time of recording, we later contacted all female speakers and confirmed that none had used any hormonal contraceptives in the 2 months prior to the recording session.

### 3.2 Experimental procedure

Although multiple body size and vocal tract measurements would ideally be collected, height was the primary anthropometric variable measured in the present study. We therefore use “height” when referring to the observed variable and reserve “body size” for the broader construct for which height serves as a proxy when comparing our findings with previous vocal allometry studies (Titze, 1989; Fitch, 1997). Recordings were made using Praat (version 6.4.46) in the Phonetics Laboratory of the School of Liberal Arts, Guangxi University. Participants read a standardized text (the Mandarin version of The North Wind and the Sun) as well as words and sentences at a natural speaking rate. All recordings were digitized at a 44.1 kHz sampling rate, 16-bit quantization, mono channel, and saved in WAV format.

### 3.3 Methods

#### 3.3.1 Fundamental frequency

Fundamental frequency (F0) parameters were extracted from the full recording of each speaker’s reading of the Mandarin North Wind and the Sun passage using Praat’s autocorrelation method. The pitch search range was set to 75–500 Hz. Silence and voicing thresholds were adjusted to optimize the continuity of the pitch contour and minimize octave jumps and outliers. The median F0 (F0-median) was adopted as the measure of typical pitch level because it is robust against non-normal distributions and extreme values. Each speaker thus yielded a single F0-median value for subsequent statistical analyses.

#### 3.3.2 Formant frequencies and dispersion

In Praat’s formant analysis settings, the maximum number of formants was set to 4. The ceiling frequency was set to 5.5 kHz for female speakers and 5.0 kHz for male speakers to avoid tracking spurious formants.

Formant dispersion (Df) was calculated as the mean spacing between successive formants. Following Puts et al. (2011a) and Puts et al. (2011b), when four formants (F1–F4) are measured, this measure is algebraically equivalent to (F4 − F1)/3. We therefore calculated Df as (F4 − F1)/3 for each vowel. This formulation provides a compact index of formant spacing and has been used in studies of vocal-tract configuration and body-size variation. However, because the measure is determined by the lowest and highest measured formants, it may be particularly sensitive to variation in F4; therefore, Df was interpreted as an acoustic correlate of vocal-tract dimensions rather than as a direct measure of vocal-tract length or body size (Puts et al., 2011a; Puts et al., 2011b; Anikin et al., 2024).

We analyzed three corner vowels /a/, /i/, and /u/ in Mandarin extracted from isolated words. For each speaker, five tokens per vowel were selected, and the median formant values across these tokens were computed. Df was calculated separately for each vowel, allowing vowel-specific analyses.

Praat version 6.4.46 was used. For formant analysis, the Burg algorithm was applied with a window length of 0.025 s and a dynamic range of 30 dB. Formant tracks were visually inspected, and frames with tracking errors were excluded. The maximum number of formants was set to 4.

### 3.4 Statistical analysis

Bayesian models were fitted using brms (v2.23.0; Bürkner, 2017) in R (v4.5.1), with the rstan (v2.32.7) backend for MCMC sampling via the No-U-Turn Sampler (NUTS); (see Stan Development Team, 2024). For each regression coefficient, we specified a weakly informative prior Normal (0, 2.5); the intercept was assigned Normal(0, 10), and the residual standard deviation used the default half-Student-t prior, student_t (3, 0, MAD(y)). Four chains were run for 6,000 iterations each, with the first 3,000 discarded as warm-up; adapt_delta was set to 0.99. Convergence was assessed via Rhat (<1.01) and effective sample size. Model comparison was conducted using leave-one-out cross-validation (LOO) and the Watanabe–Akaike information criterion (WAIC).

### 3.5 Results

#### 3.5.1 Sex differences in F0 and Df

F0 has shown a pronounced sex difference. The posterior mean difference between females and males was 97.5 Hz (95% CrI [77.4, 117.3]), corresponding to a large standardized effect (Cohen’s *d* = 4.02, 95% CrI [2.98, 5.06]). The posterior probability that females had higher F0 than males was 1.000. Thus, F0 showed a clear and substantial degree of sexual dimorphism.

Sex differences in Df varied across vowels. For /a/, the estimated difference was 66.0 Hz (95% CrI [−2.3, 136.3]; *d* = 0.75, 95% CrI [−0.09, 1.56]), with the credible interval for the standardized difference overlapping zero. In contrast, the sex differences were larger for /u/ (*d* = 1.83, 95% CrI [0.96, 2.68]) and /i/ (*d* = 2.07, 95% CrI [1.18, 2.94]), with both credible intervals clearly above zero. The corresponding posterior probabilities that females had higher Df than men were 1.000 for both /u/ and /i/ (Table 1).

**Table 1:** Sex differences in acoustic parameters (raw scale and standardized Cohen’s *d*).

Parameter	Difference (Hz)	95% CrI	Cohen’s *d* (median)	95% CrI	*P* (females > males)
F0	97.5	[77.4, 117.3]	4.02	[2.98, 5.06]	1.000
/a/ Df	66.0	[−2.3, 136.3]	0.75	[−0.09, 1.56]	0.971
/u/ Df	153.8	[83.7, 225.0]	1.83	[0.96, 2.68]	1.000
/i/ Df	196.2	[117.7, 271.9]	2.07	[1.18, 2.94]	1.000

The difference (Hz) is the posterior mean difference between females and males (females − males), estimated at mean height from the full raw-scale model (parameter ~ height × Sex, weakly informative prior). Cohen’s d is derived from the corresponding standardized model (parameter ~ height_z × Sex) as the posterior distribution of the standardized mean difference. P(females > males) is the posterior probability that the raw-scale difference exceeds zero. CrI = 95% credible interval. The CrI for /a/ Df crosses zero, indicating weaker evidence for a sex difference in this parameter.

#### 3.5.2 Within-sex associations between height and acoustic parameters

We next examined whether the associations between height and the acoustic parameters persisted when males and females were analyzed separately. F0 showed little evidence of a within-sex association with height in either sex. In males, the posterior median correlation was *r* = −0.10 (95% CrI [−0.43, 0.22]), while in females it was *r* = −0.21 (95% CrI [−0.52, 0.10]). In both cases, the credible intervals overlapped zero (Table 2).

**Table 2:** Within-sex correlations between height and acoustic parameters (raw scale).

Parameter	Sex	r (median)	95% CrI	P(r < 0)
F0	males	−0.10	[−0.43, 0.22]	0.732
F0	females	−0.21	[−0.52, 0.10]	0.908
/a/ Df	males	−0.27	[−0.59, 0.05]	0.956
/a/ Df	females	−0.33	[−0.65, −0.02]	0.982
/u/ Df	males	−0.27	[−0.59, 0.04]	0.957
/u/ Df	females	−0.03	[−0.35, 0.30]	0.562
/i/ Df	males	−0.23	[−0.55, 0.09]	0.921
/i/ Df	females	−0.20	[−0.52, 0.12]	0.893

Correlation coefficients (*r*) were derived from within-sex subset regressions (parameter ~ height, raw scale, weakly informative prior) by rescaling the posterior slope: r = β × SD(height) / SD(parameter). With the exception of /a/ Df in females, all 95% credible intervals cross zero, indicating that within-sex associations between height and the acoustic parameters are generally weak and imprecisely estimated.

Df has shown a more heterogeneous pattern across vowels and sexes. For /a/, the correlation was negative in both males (*r* = −0.27, 95% CrI [−0.59, 0.05]) and females (*r* = −0.33, 95% CrI [−0.65, −0.02]), but the credible interval excluded zero only in females. The /u/ Df correlations were −0.27 (95% CrI [−0.59, 0.04]) in males and −0.03 (95% CrI [−0.35, 0.30]) in females. For /i/, the corresponding estimates were −0.23 (95% CrI [−0.55, 0.09]) in males and −0.20 (95% CrI [−0.52, 0.12]) in females. Thus, among the within-sex correlations, the clearest evidence for a height-related association was observed for female /a/ Df, whereas the remaining credible intervals overlapped zero (Table 2).

#### 3.5.3 Within-sex regression slopes

To characterize these associations on the original acoustic scale, we estimated within-sex regression slopes of each acoustic parameter on height. F0 slopes were negative in both females (−0.73 Hz/cm, 95% CrI [−2.09, 0.68]) and males (−0.94 Hz/cm, 95% CrI [−2.18, 0.32]), but both credible intervals overlapped zero.

For Df, the strongest negative slopes were observed for /a/. The estimated slope was −5.58 Hz/cm (95% CrI [−9.92, −1.19]) in females and −5.92 Hz/cm (95% CrI [−9.91, −1.91]) in males. A negative slope was also estimated for /i/ Df in males (−4.88 Hz/cm, 95% CrI [−9.26, −0.53]). By contrast, the credible intervals for /u/ Df in females (−2.44 Hz/cm, 95% CrI [−6.75, 1.90]) and males (−3.42 Hz/cm, 95% CrI [−7.37, 0.67]), as well as for /i/ Df in females (−3.74 Hz/cm, 95% CrI [−8.62, 0.96]), overlapped zero (Table 3).

**Table 3:** Within-sex Bayesian regression slopes of acoustic parameters on height (raw scale).

Parameter	Sex	Slope (mean)	95% CrI lower	95% CrI upper	P(slope < 0)
F0 (Hz/cm)	females	−0.73	−2.09	0.68	0.851
F0 (Hz/cm)	males	−0.94	−2.18	0.32	0.933
/a/ Df (Hz/cm)	females	−5.58	−9.92	−1.19	0.993
/a/ Df (Hz/cm)	males	−5.92	−9.91	−1.91	0.998
/u/ Df (Hz/cm)	females	−2.44	−6.75	1.90	0.867
/u/ Df (Hz/cm)	males	−3.42	−7.37	0.67	0.953
/i/ Df (Hz/cm)	females	−3.74	−8.62	0.96	0.939
/i/ Df (Hz/cm)	males	−4.88	−9.26	−0.53	0.986

Slopes were estimated from within-sex subset models (parameter ~ height, raw scale, weakly informative prior; *N* = 40 per sex) and are expressed in Hz per centimeter of height. 95% credible intervals exclude zero only for /a/ Df (both sexes) and /i/ Df (males).

#### 3.5.4 Predictive contribution of sex

We then compared three Bayesian models for each acoustic parameter: a height-only model (M1), a model including height and sex (M2), and a sex-only model (M3). For F0, adding sex to the height-only model substantially improved predictive performance. The median Bayesian *R*^2^ increased from 0.646 (95% CrI [0.546, 0.704]) in M1 to 0.846 (95% CrI [0.809, 0.863]) in M2, and the improvement in expected log predictive density was ΔELPD/SE = 1.92. The sex-only model performed similarly to the height-plus-sex model (ΔELPD/SE = 1.91 relative to M1), indicating that most of the predictive information captured by the combined model was associated with sex rather than height.

For Df, the additional predictive contribution of sex was more limited. For /a/ Df, adding sex increased the median R^2^ only modestly, from 0.447 to 0.475, with ΔELPD/SE = 0.06. For /u/ and /i/ Df, the corresponding ΔELPD/SE values were 0.84 and 1.12, respectively. Thus, in contrast to F0, adding sex provided only limited additional predictive improvement for Df, although the magnitude of this improvement varied across vowels (Table 4).

**Table 4:** Bayesian model comparison for standardized acoustic parameters.

Parameter	Model	R^2^ (median)	R^2^ 95% CrI	ELPD	ΔELPD (vs M1)	ΔELPD/SE
F0	M1: height_z	0.646	[0.546, 0.704]	−74.70	—	—
F0	M2: height_z + Sex	0.846	[0.809, 0.863]	−43.65	31.05 (SE 16.19)	1.92
F0	M3: Sex only	0.844	[0.807, 0.860]	−43.62	31.08 (SE 16.25)	1.91
/a/ Df	M1: height_z	0.447	[0.300, 0.547]	−92.10	—	—
/a/ Df	M2: height_z + Sex	0.475	[0.334, 0.570]	−91.60	0.50 (SE 8.11)	0.06
/a/ Df	M3: Sex only	0.419	[0.264, 0.527]	−94.59	−2.49 (SE 9.51)	−0.26
/u/ Df	M1: height_z	0.483	[0.347, 0.575]	−89.44	—	—
/u/ Df	M2: height_z + Sex	0.585	[0.468, 0.655]	−81.77	7.67 (SE 9.09)	0.84
/u/ Df	M3: Sex only	0.573	[0.451, 0.648]	−81.77	7.67 (SE 9.06)	0.85
/i/ Df	M1: height_z	0.563	[0.438, 0.639]	−82.83	—	—
/i/ Df	M2: height_z + Sex	0.673	[0.579, 0.725]	−72.43	10.40 (SE 9.27)	1.12
/i/ Df	M3: Sex only	0.659	[0.564, 0.714]	−73.29	9.55 (SE 9.83)	0.97

R^2^ is the median and 95% credible interval of the Bayesian R^2^ posterior distribution (computed separately for each model with bayes_R2(fit, summary = FALSE)). Model comparison is based on the expected log predictive density (ELPD) from Pareto-smoothed importance-sampling leave-one-out cross-validation (PSIS-LOO, moment matching); WAIC values are omitted here but were consistent with the LOO-based ranking. ΔELPD/SE values with |ΔELPD/SE| < 1 are treated as weak evidence for a difference between models, following the same threshold used in the prior-sensitivity analysis. Adding Sex to the height-only model produced a credible improvement in fit for F0 (|ΔELPD/SE| ≈ 1.9) but only weak evidence for improvement in the three formant-dispersion measures (/a/, /u/, /i/ Df; |ΔELPD/SE| < 1.2), for which height alone already explains a comparable proportion of variance.

#### 3.5.5 Relative height sensitivity of Df and F0

Finally, we directly compared the posterior height slopes of Df and F0 on the standardized scale. The comparison was expressed as the posterior probability that the Df slope was more negative than the corresponding F0 slope within each sex. (Figures 1, 2).

**Figure 1:**
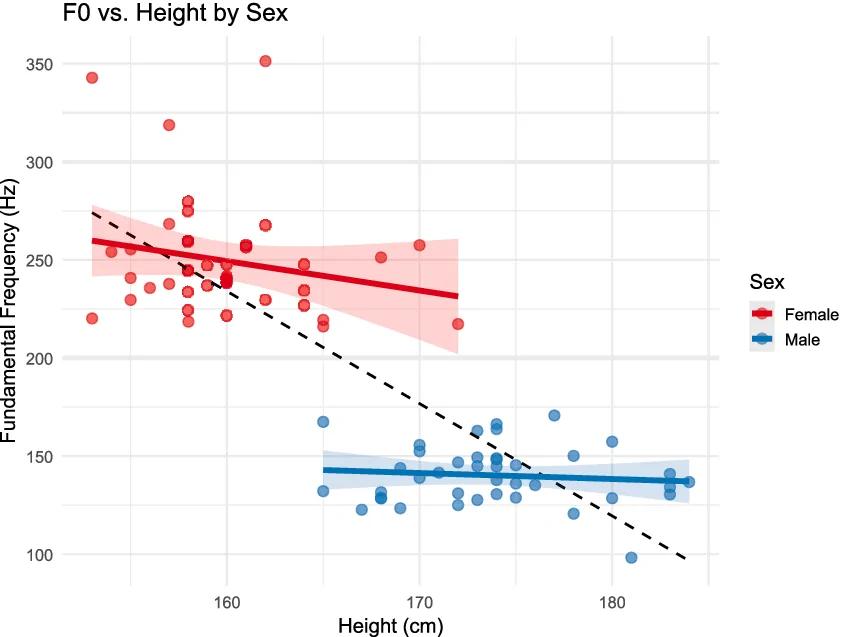
F0 vs. height by sex.

**Figure 2:**
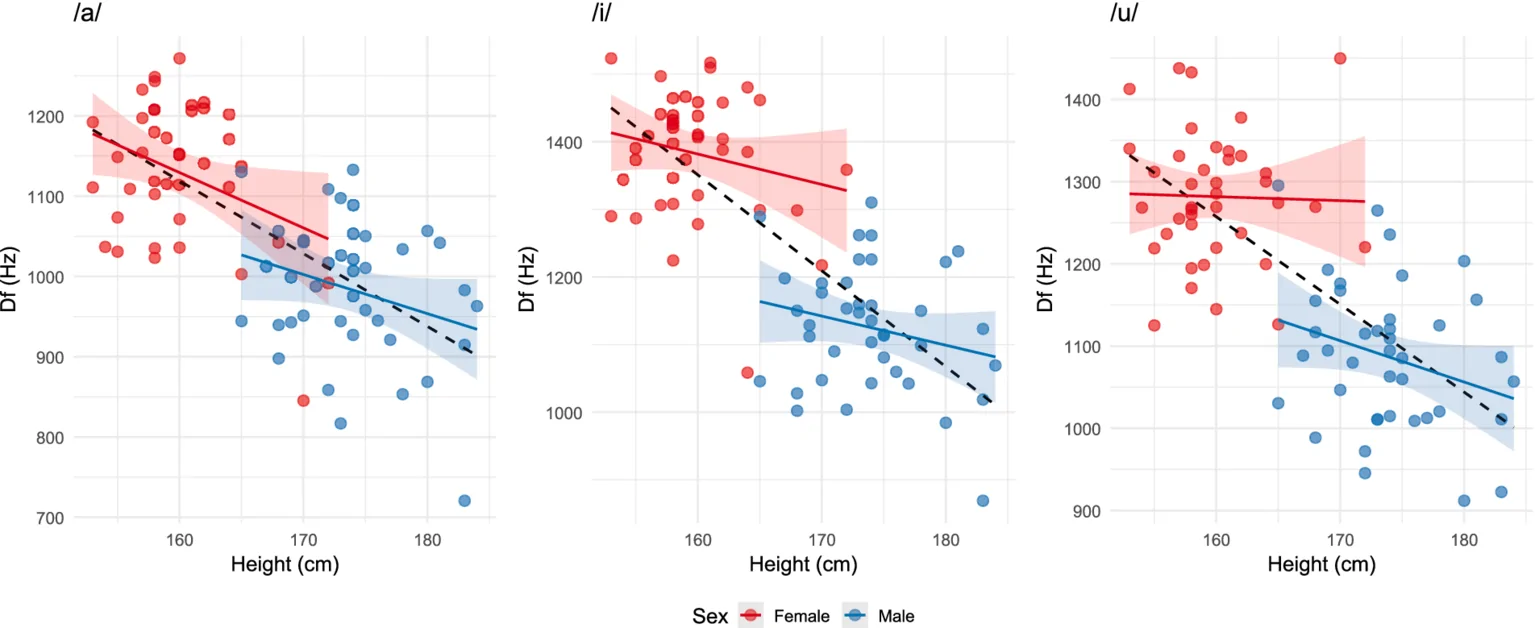
Df vs. height by sex.

For /a/, this probability was 0.866 in females and 0.913 in males, providing relatively strong evidence that the Df height slope was more negative than the F0 slope in both sexes. For /u/, the probability was 0.236 in females but 0.906 in males, indicating a sex-dependent pattern. For /i/, the corresponding probabilities were 0.554 in females and 0.826 in males. Thus, the posterior comparison suggested a generally stronger negative height slope for Df than for F0 in males across all three vowels, whereas this pattern was evident only for /a/ Df in females (Table 5).

**Table 5:** Posterior comparison of height slopes for formant dispersion (Df) versus F0 (standardized scale).

Parameter	P(Df slope < F0 slope) — female	P(Df slope < F0 slope) — male
/a/ Df	0.866	0.913
/u/ Df	0.236	0.906
/i/ Df	0.554	0.826

Values are posterior probabilities, computed from the standardized full models, that the height slope for the given formant-dispersion (Df) measure is more negative than the height slope for F0, within each sex. In males, this probability is consistently high (0.83–0.91) across all three vowels, suggesting formant dispersion may be more sensitive to height than F0 is. In females, only /a/ Df shows a comparable pattern; evidence is weak for /u/ Df and /i/ Df.

#### 3.5.6 Summary of module 1 findings

Overall, F0 showed a strong and consistent sex difference but little evidence of a within-sex association with height. Df also showed sex differences, although their magnitude varied substantially across vowels. Within sexes, the evidence for a height-related association with Df was limited and vowel-specific, with the clearest correlation observed for female /a/ Df. Raw-scale slope estimates showed negative relationships for /a/ Df in both sexes and for /i/ Df in males. Model comparison further indicated that sex contributed substantial predictive information for F0 but provided only limited additional predictive improvement for Df. Finally, posterior slope comparisons suggested that Df tended to show a more negative height slope than F0 in males, whereas this pattern was evident primarily for /a/ in females.

## 4 Module 2: just-noticeable difference (JND) perception experiment

### 4.1 Experimental materials

To ensure that the acoustic parameter manipulations in the subsequent perception experiment were perceptually equivalent and discriminable, we first measured the just-noticeable difference (JND) for fundamental frequency (F0) and formant dispersion (Df). This pilot experiment recruited 20 participants (10 males, 10 females). All participants reported no speech or hearing disorders. Using a same–different discrimination task, participants listened to an original stimulus and a modified stimulus. We manipulate the voice samples “天空是蓝的, 水是绿的” (“The sky is blue, the water is green”; one male and one female speaker from Module 1) in Praat, applying continuous parameter adjustments with equal physical step sizes. F0 was modulated in units of Equivalent Rectangular Bandwidth (ERB), while Df was modulated as a percentage change (Puts, 2010). Specifically, F0 was adjusted over 18 steps (nine increases, nine decreases) with a step size of 0.05 ERB, yielding a total adjustment range of ±0.05 to ±0.45 ERB; Df was adjusted over 18 steps with a step size of 1%, yielding a total adjustment range of ±2 to ±10%. All manipulations were verified by measurement in Praat, and any sample failing to meet the precision requirement was reprocessed. To exclude mutual interference between parameters, we check whether the other parameter changed unintentionally after each single-parameter adjustment. Specifically, after adjusting Df, we inspect F0’s shift, and vice versa. If any unintended deviation occurs, the parameter is corrected back.

### 4.2 Experimental procedure

The experiment was hosted on the Credamo^2^. All participants completed the task individually on a laboratory computer inside a sound-attenuated booth at the School of Literature Phonetics Laboratory, under the supervision of an experimenter. All participants used the same model of headphones (Sony MDR-7506), and playback was presented at a fixed comfortable level. On each trial, participants listened to an original stimulus and a modified stimulus, which was adjusted by the same physical parameter (presentation order randomized), and then judged whether the two stimuli sounded “same” or “different.” Responses from all 20 participants across 72 trials were recorded.

### 4.3 Data analysis and results

For each condition, we began by calculating the proportion of trials labelled “different” at every change level. Psychometric functions were then fitted to these data using logistic regression, and we took the change level corresponding to 85% “different” responses as the just-noticeable difference (JND). This threshold was chosen to ensure that the ±1 JND manipulations in Module 3 would be clearly discriminable, consistent with the use of elevated criteria in auditory discrimination tasks. This conservative high-threshold criterion minimizes the risk that ambiguous discrimination near threshold would contaminate the subsequent social judgment ratings.

To estimate 95% confidence intervals around the JND, we applied bootstrap resampling (2,000 replicates). Despite the moderate sample size (*N* = 20), bootstrap resampling was used to quantify sampling uncertainty around the estimated thresholds (Efron and Tibshirani, 1994), and the resulting confidence intervals were sufficiently narrow to inform the manipulation magnitudes in Module 3. We also used McFadden’s pseudo-R^2^ as a measure of model fit.

Across all conditions, the logistic models performed well, with pseudo-R^2^ values falling between 0.58 and 0.86.

For formant dispersion (Df), JND values ranged from 4.33 to 8.07 change levels. The least sensitive condition was Female_Df_Increase (JND = 8.07, 95% CI [6.77, 9.65]).

For the fundamental frequency (ERB), JND values ranged from 5.69 to 8.06 change levels. The least sensitive condition was Female_ERB_Increase (JND = 8.06, 95% CI [7.13, 9.07]). (Figure 3).

**Figure 3:**
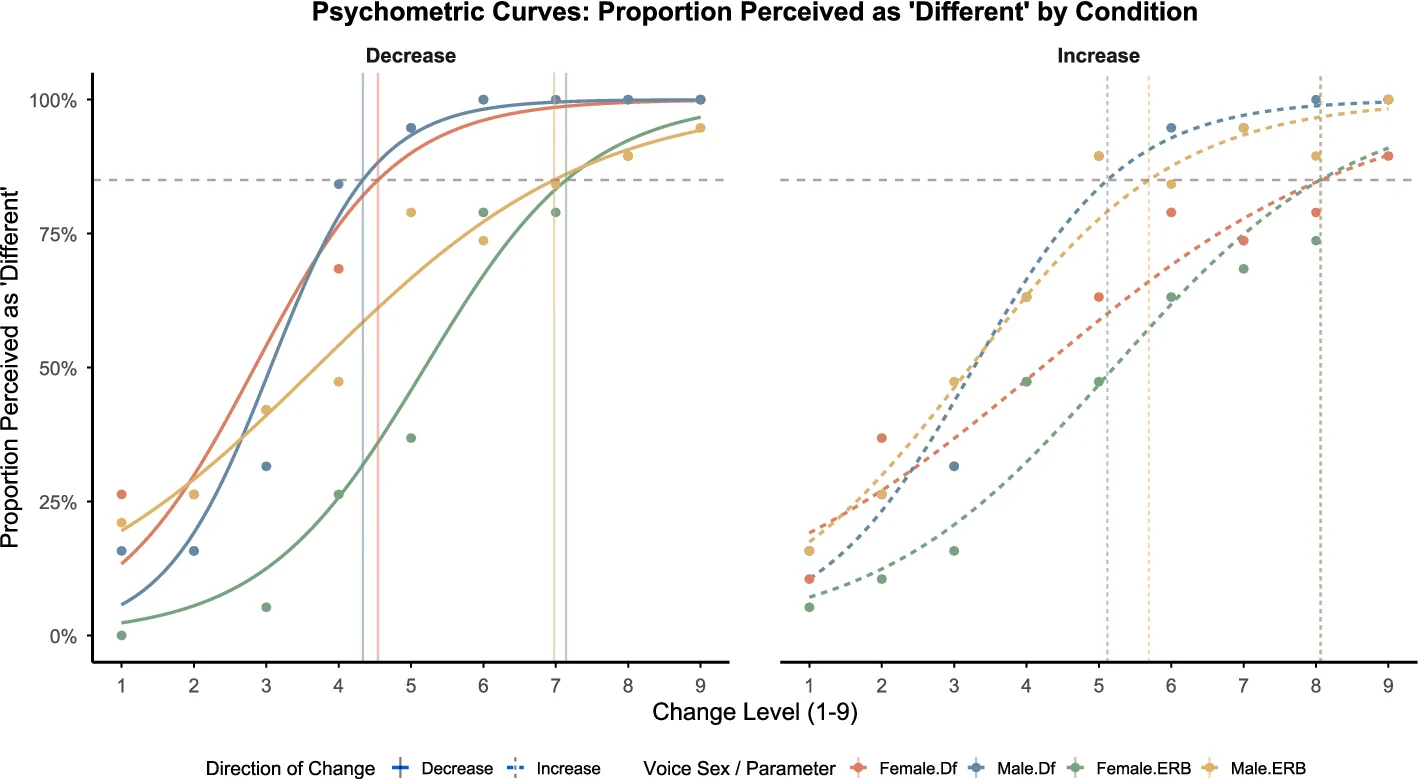
Proportion perceived as ‘different’ by condition.

To ensure that the parameter manipulations in the main study produced clear and stable perceptual effects, we adopted a conservative approach: for each parameter, we selected the condition requiring the largest change (i.e., the highest JND) as the basis for setting the universal perceptual threshold. Accordingly, the adjustment magnitude for Df was set to 8.07 change levels, and that for F0 (ERB) was set to 8.06 change levels. We therefore used the largest point-estimate threshold observed for each acoustic parameter as a conservative common manipulation magnitude, which allowed for individual perceptual variability and ensured that the vast majority of participants could stably detect the change. Accordingly, Df was set to 8.07%, and F0 was set to 0.40 ERB. Detailed data are provided in the Supplementary materials.

## 5 Module 3: perceptual rating experiment

### 5.1 Materials

Two trained phoneticians independently listened to each recording of the target sentence (“天空是蓝的, 水是绿的”) and rated two aspects: (a) segmental accuracy – whether any consonant or vowel deviated from standard Mandarin pronunciation to a degree that could cause phonemic confusion (e.g., /n/−/l/ merger, /ɻ/−/l/ merger, or other non-standard articulations); and (b) prosodic naturalness – whether the utterance sounded noticeably unnatural in intonation, rhythm, or stress (e.g., a flat or exaggerated pitch contour unrelated to the speaker’s habitual voice). Any speaker who received a rating of “clearly deviant” from both phoneticians on either dimension was excluded from further analysis. Inter-rater agreement was stable (Cohen’s κ = 0.92). Based on this criterion, four speakers were excluded: two males (M16, M18) due to /n/−/l/ or /ɻ/−/l/ merger, and two females (W7, W20) due to /n/−/l/ merger and abnormal intonation, respectively. The final speaker pool consisted of 38 males and 38 females (N = 76).

To avoid excessively long experimental sessions and participant fatigue in Module 3, five male and five female speakers were randomly selected without replacement from this eligible pool using R (set.seed (20260315); sample (*n* = 5)). Independent-samples t-tests confirmed that the selected subsample did not differ significantly from the remaining pool in mean F0 (males: *t* = 0.12, *p* = 0.91; females: *t* = 0.08, *p* = 0.94), indicating adequate representativeness.

All 10 selected speakers produced the same sentence (“天空是蓝的, 水是绿的”). Each recording was normalized in intensity to 70 dB. For each original (baseline) voice, four manipulated versions were created by shifting either F0 or Df by ±1 just-noticeable difference (JND), resulting in a “masculinized” (lower F0 or lower Df) and a “feminized” (higher F0 or higher Df) version, while keeping all other acoustic features unchanged. This yielded a total of 50 stimuli (2 sexes × 5 speakers × 5 levels: baseline, Df-up, Df-down, F0-up, F0-down).

### 5.2 Participants

Ninety participants (45 males, 45 females) were recruited. All were native speakers of Northern Mandarin dialects, fluent in Standard Mandarin, with no self-reported hearing or speech disorders. None of the participants in Module 3 had previously taken part in any similar experiment. All participants self-identified as heterosexual and single.

### 5.3 Experimental procedure

Opposite-sex perception: Female and male participants listened to audio recordings of opposite-sex speakers, including both the original voice and two manipulated versions contrasting in pitch (F0) and formant dispersion (Df). After each stimulus presentation, participants rated its attractiveness on a 7-point Likert scale (1 = “very unattractive,” 4 = “neutral,” 7 = “very attractive”) to test the hypothesis regarding opposite-sex attractiveness.

Same-sex perception: In the female participant task, participants listened to female voices processed under the same conditions and rated each voice on “how flirtatious it sounded toward males” (1 = “not at all flirtatious,” 4 = “neutral,” 7 = “very flirtatious”), aiming to indirectly assess intrasexual competition threat through perceived flirtatiousness. In the male participant task, participants listened to male voices and rated each voice on “aggressiveness” (1 = “not at all aggressive,” 4 = “neutral,” 7 = “very aggressive”) to test the hypothesis regarding aggression perception.

All experimental procedures included a practice session. The presentation order of recordings and stimuli was randomized. The specific rating instructions were as follows:

Attractiveness: “In a short-term dating scenario, how attractive do you find this voice?”

Aggressiveness: “How aggressive do you think the owner of this voice is?”

Flirtatiousness: “Please judge how much this voice sounds like it is actively making a romantic invitation or flirting in a dating context.”

### 5.4 Results

For the repeated-measures rating data in Module 3, we employed linear mixed-effects models (LMMs) within a frequentist framework. This approach is standard for analyzing crossed random effects of participants and stimuli in factorial perceptual experiments (Baayen et al., 2008), and allows for straightforward calculation of Cohen’s d effect sizes from the estimated marginal means. Module 1 used Bayesian regression to quantify posterior uncertainty in the acoustic associations (Knill and Pouget, 2004), whereas Module 3 used linear mixed-effects models because the rating data involved crossed repeated measures of participants and stimuli.

To examine the relative importance of formant dispersion (Df) and fundamental frequency (F0) in the social perception of voices, we construct linear mixed models (LMMs) separately for each of the four evaluator–stimulus sex combinations. Parameter type (Df vs. F0), JND change level (−1, 0, +1), and their interaction were entered as fixed effects. Each stimulus was derived from one of 10 unique base speakers, and speaker identity was included as a random intercept. The model results are summarized in Table 6.

**Table 6:** Perceptual rating slopes and effect sizes.

Evaluator group	Dependent variable	Df slope	Df effect size (Cohen’s *d*)	ERB slope	ERB effect size (Cohen’s *d*)	Df effect size > ERB (%)
Male eval Male	Aggressiveness	−2.818	3.782	−1.738	2.146	76.3%
Male eval Female	Attractiveness	2.596	3.372	1.498	2.079	62.2%
Female eval Male	Attractiveness	−2.636	3.228	−1.773	2.892	11.6%
Female eval Female	Flirtatiousness	2.298	3.071	1.533	1.928	59.3%

#### 5.4.1 Comparison of perceptual effect sizes: Df vs. F0

The main effect of change level was significant across all four evaluator–stimulus conditions (all *p* < 0.001), and the interaction between parameter type and change level was also significant in every condition (all *p* < 0.001). Simple-slope analyses showed that within each rating task, the estimated effect of Df manipulation was larger than that of F0 manipulation. (Table 5). The corresponding Cohen’s d values were 3.782 versus 2.146 for male-evaluated male aggressiveness, 3.372 versus 2.079 for male-evaluated female attractiveness, 3.228 versus 2.892 for female-evaluated male attractiveness, and 3.071 versus 1.928 for female-evaluated female flirtatiousness (Df vs. F0, respectively; Table 6).

Thus, when acoustic changes were equated in perceptual terms using JND units, Df produced larger estimated effects on social ratings than F0 across all four rating tasks.

#### 5.4.2 Sex-asymmetric pattern: intrasexual competition vs. intersexual attraction

Across the four sex-combination groups, a clear pattern emerged: (1) In intersexual attraction, female listeners rated masculinized voices (−1 JND) as more attractive, whereas male listeners preferred feminized voices (+1 JND). (2) In intrasexual competition, male listeners rated masculinized voices as more aggressive, while female listeners rated feminized voices as more flirtatious.

Notably, the effect sizes of Df and F0 showed a systematic sex asymmetry (Figure 4 and Table 6). For male evaluators, Df had a larger effect on judgements of intrasexual aggression (Df *d* = 3.782 vs. F0 *d* = 2.146) and on opposite-sex attractiveness (Df *d* = 3.372 vs. F0 *d* = 2.079), indicating that Df consistently outweighed F0 across both judgment domains. For female evaluators, the reverse held: Df’s effect was larger for attractiveness judgments of opposite-sex voices (Df *d* = 3.228 vs. F0 *d* = 2.892) and for intrasexual flirtatiousness (Df *d* = 3.071 vs. F0 *d* = 1.928), again demonstrating Df’s superior perceptual salience regardless of the specific social judgment.

**Figure 4:**
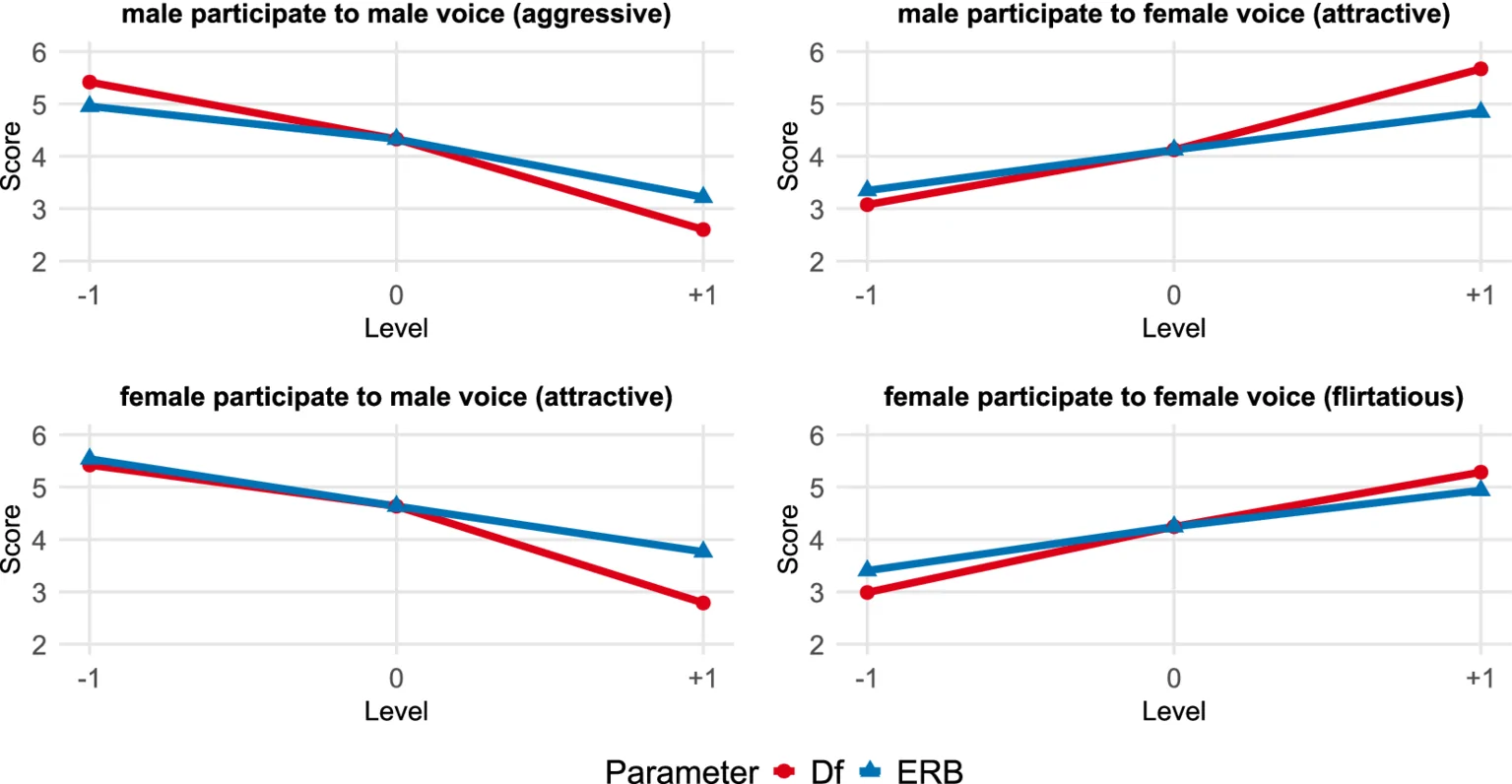
Perception rating score.

Within each judgment domain, Df consistently showed a larger effect size than F0 for both male and female evaluators. This pattern held across intrasexual competition judgments (aggressiveness for males, flirtatiousness for females) and intersexual attraction judgments (attractiveness for both sexes). The fact that the estimated effect of Df is greater than that of F0 is consistent with the possibility that Df provides information that listeners treat as socially relevant. However, because the present study did not directly test listeners’ ability to infer height from Df, this pattern should not be interpreted as evidence that Df is an honest signal of body size. The fact that Df and F0 carry different weights across sexes and contexts—serving as acoustic vehicles for these two classes of social signals. The consistent perceptual sensitivity to Df and F0 across rating tasks suggests that both acoustic dimensions can influence socially relevant voice judgments. Whether this perceptual sensitivity reflects adaptations shaped by sexual selection remains an open question.

## 6 Discussion

### 6.1 F0 and Df show different relationships with sex and height

The present study provides evidence that F0 and Df contribute differently to human vocal sexual dimorphism. F0 showed a very large sex difference (Cohen’s *d* = 4.02, 95% CrI [2.98, 5.06]), but little evidence of a within-sex association with height in either males or females. This pattern is consistent with previous evidence that human vocal dimorphism cannot be explained by simple body-size scaling. In particular, the human female-to-male F0 ratio is substantially larger than the corresponding height ratio, suggesting that the magnitude of vocal dimorphism exceeds that expected from body size alone (Rendall et al., 2005). The present within-sex analysis extends this observation by showing that individual differences in height explain little of the variation in F0 once sex is considered.

This pattern is also compatible with the developmental basis of F0 dimorphism. Male puberty involves substantial androgen-dependent changes in laryngeal and vocal-fold morphology, producing a marked reduction in F0 (Fant, 1960; Fitch and Giedd, 1999; Barbier et al., 2015; Styne et al., 1998). The unusually large sex difference in human F0, relative to body-size differences, may therefore reflect sex-specific developmental effects rather than simple proportional scaling of vocal anatomy. Comparative evidence likewise suggests that human vocal dimorphism departs from the more proportional acoustic allometry observed in some non-human primates (Puts et al., 2016).

Df showed a different and more heterogeneous pattern. Sex differences were substantial for /u/ and /i/, but weaker for /a/. Within sexes, the clearest standardized association was observed for female /a/ Df (*r* = −0.33, 95% CrI [−0.65, −0.02]), whereas the corresponding association in males was negative but uncertain. Associations for /u/ and /i/ were similarly uncertain. Regression slopes nevertheless showed negative relationships for /a/ Df in both sexes and for /i/ Df in males. Taken together, these results provide limited and vowel-specific evidence that Df covaries with height within sex, rather than evidence for a general Df–height relationship (Fitch and Giedd, 1999; Puts et al., 2011a; Puts et al., 2011b).

This distinction is important because formant dispersion has a clearer anatomical basis than F0 as a potential index of vocal-tract dimensions. Vocal-tract length is closely related to formant structure, and previous comparative work has demonstrated relationships between formant dispersion and body size in primates. However, the present results indicate that such a relationship is not uniform across vowels or sexes. Thus, Df may retain some information related to vocal-tract dimensions and height, although this relationship appears to be vowel- and sex-dependent.

### 6.2 Sex differences do not necessarily indicate body-size signaling

The contrasting patterns of F0 and Df highlight an important distinction between sexual dimorphism and body-size signaling. A parameter can differ strongly between males and females without necessarily providing reliable information about height among individuals of the same sex. F0 provides the clearest example: its sex difference was very large, while its within-sex relationship with height was weak. This finding is consistent with earlier work showing that human F0 dimorphism is disproportionately large relative to sex differences in height (Rendall et al., 2005).

Df provides a more nuanced case. Although Df also differed between the sexes, its within-sex associations with height were modest and vowel-dependent. This pattern suggests that the sex difference in Df should not be interpreted simply as a consequence of differences in overall body size. Rather, sex-specific anatomical development and vocal-tract configuration may contribute alongside general body-size variation.

The model-comparison results reinforce this distinction. Adding sex substantially improved predictive performance for F0, whereas the additional predictive benefit of sex for Df was limited and varied across vowels. Thus, sex accounted for a particularly large proportion of the predictive structure of F0, whereas Df showed a less uniform relationship with sex and height. These findings suggest that the two acoustic dimensions may carry partly distinct information rather than constituting redundant indicators of a single underlying body-size trait.

### 6.3 Implications for sexual selection and vocal signaling

The present findings are relevant to, but do not by themselves establish, a sexual-selection account of human vocal dimorphism. Sexual dimorphism is often associated with sexual selection in primates, particularly where sexually differentiated traits influence mate choice or intrasexual competition (Plavcan, 2012; Darwin, 2010; Fitch and Hauser, 1995). Human voices are potentially relevant in this context because acoustic variation can affect judgments of attractiveness, dominance, and aggressiveness (Puts et al., 2006, 2007; Feinberg et al., 2005, 2008; Bruckert et al., 2010; O’Connor et al., 2014; Quené et al., 2021). In males, lower F0 and more closely spaced formants have been associated with perceptions of greater competitive ability, and vocal characteristics have also been related to upper-body strength and testosterone levels (Puts, 2005; Morton, 1977; Ohala, 1995). Female vocal characteristics similarly influence judgments of attractiveness and femininity (Feinberg et al., 2008).

The present acoustic results refine this framework by suggesting that F0 and Df may not convey the same type of information. F0 appears to be strongly differentiated by sex but provides little within-sex information about height. Df, in contrast, shows more limited but detectable height-related variation under some conditions. If listeners use these cues in social evaluation, the two dimensions could therefore contribute differently to judgments relevant to mate choice or competition.

This interpretation is particularly relevant to the distinction between signal magnitude and signal reliability. A larger sex difference does not necessarily imply greater perceptual or evolutionary importance. Previous studies have shown that relatively small manipulations of F0 and formant frequencies can influence attractiveness and social judgments (Feinberg et al., 2005, 2008; Zheng et al., 2020). The present findings suggest that the informational value of an acoustic cue may depend not simply on the magnitude of its sexual dimorphism but also on the extent to which it covaries with stable characteristics of the speaker.

However, the present data do not establish that Df is an “honest signal” of height. The within-sex Df–height associations were limited and vowel-specific, and the study did not directly measure whether listeners accurately infer height from Df. Moreover, an association between an acoustic feature and body size is not sufficient to demonstrate that the feature evolved through sexual selection. The more conservative interpretation is that Df may provide some size-related information that could potentially contribute to social signaling, a possibility that should be tested directly in future perceptual and comparative work.

### 6.4 Why might F0 and Df have different signaling functions?

The different acoustic patterns may reflect differences in the anatomical and developmental determinants of F0 and Df. F0 is strongly affected by vocal-fold length, mass, and tension, all of which undergo substantial sex-specific changes during puberty. Df, by contrast, is primarily determined by the configuration and length of the vocal tract and therefore may be more directly related to vocal-tract dimensions (Puts et al., 2011a; Puts et al., 2011b; Fitch and Reby, 2001).

This anatomical distinction provides a plausible explanation for why F0 can exhibit extreme sexual dimorphism without a strong within-sex relationship with height, whereas Df may retain some size-related variation. It also fits comparative evidence suggesting that human F0 dimorphism is unusually pronounced relative to body-size dimorphism (Rendall et al., 2005; Puts et al., 2016).

From an evolutionary perspective, these differences raise the possibility that F0 and Df have undergone partially distinct selection pressures. F0 may have become particularly differentiated through sex-specific developmental and social processes, whereas Df may preserve more direct information about vocal-tract dimensions. Such differentiation would be compatible with a multi-cue signaling system in which different acoustic parameters provide partially independent information about the speaker.

Importantly, this remains an evolutionary interpretation rather than a demonstrated mechanism. The present study is cross-sectional and does not provide direct evidence of selection, heritability, reproductive consequences, or ancestral states. Consequently, the results are best interpreted as identifying acoustic patterns that are compatible with differentiated signaling functions, rather than as demonstrating that either F0 or Df is an adaptation produced by sexual selection.

### 6.5 Limitations and future directions

Several limitations should be considered. First, the sample size was modest (40 males and 40 females), which limits precision for relatively weak within-sex associations. The uncertainty surrounding several Df–height estimates therefore warrants replication in larger samples. Our participant samples (both speakers and raters) consisted exclusively of young, heterosexual, cisgender Chinese university students. Therefore, our findings may not generalize to other age groups, cultures, or sexual/gender identities. Further research is needed to test the universality of these patterns.

Second, height was used as the primary proxy for body size. Although this facilitates comparison with previous vocal-allometry studies (Fitch, 1997), height does not capture all dimensions of body morphology. Future studies should incorporate additional anthropometric and anatomical measures, particularly vocal-tract length and upper-body dimensions.

Third, the vowel-specific pattern of Df suggests that its relationship with body size may depend on phonetic context. Further work should therefore determine whether the observed differences across /a/, /u/, and /i/ reflect articulatory configuration, measurement properties, or genuine differences in anatomical scaling.

Fourth, menstrual cycle phase was not recorded for female participants in the perceptual experiment (Module 3). Given that hormonal fluctuations can influence perceptual judgments (Pipitone and Gallup, 2008), this omission represents a limitation that should be addressed in future studies.

Finally, acoustic association alone cannot establish signaling function or sexual selection. The perceptual results in later modules provide complementary evidence that F0 and Df can influence social judgments, but such perceptual effects should not be equated directly with evolutionary adaptation. Stronger conclusions will require convergent evidence from acoustic production, perceptual inference, anatomy, development, comparative biology, and ideally fitness-relevant social outcomes.

## 7 Conclusion

Overall, the present findings indicate that human vocal sexual dimorphism is not adequately explained by a simple body-size scaling mechanism. F0 showed strong sex differentiation but little evidence of a within-sex relationship with height, whereas Df showed substantial but vowel-dependent sex differences and limited evidence of height-related variation within sex. The two acoustic parameters therefore appear to have partly differentiated relationships with sex and body size.

These findings are consistent with the possibility that F0 and Df contribute different types of information to human vocal signaling. In particular, Df may retain some information related to vocal-tract and body-size variation, whereas F0 appears to function more strongly as a marker of sex-specific vocal development. When integrated with evidence that listeners respond to variation in both parameters, these findings provide a plausible basis for investigating differentiated signaling functions in human vocal sexual dimorphism. They do not, however, by themselves establish that Df is an honest signal or that either acoustic feature is an adaptation resulting from sexual selection.

## Data Availability

The original contributions presented in the study are included in the article/Supplementary material, further inquiries can be directed to the corresponding author.

## Footnotes

1 In this article, “sex” refers to the binary sex categories, whereas “gender” refers to gender identity or expression. We distinguish these terminologies explicitly in the Method section.

2 Credamo (https://www.credamo.com) is a web-based platform designed for behavioral science experiments and survey research. It supports multimedia stimuli presentation, randomized trial sequencing, and automatic data collection.
