# Supplementary material for: Acoustic and perceptual evidence relevant to sexual selection in human voice dimorphism
Source: Front Psychol. 2026 Sep 30;17:1938866. doi: 10.3389/fpsyg.2026.1938866 (PMC13662013; doi:10.3389/fpsyg.2026.1938866)
Supplement: Supplementary file 1 [file Supplementary_file_1.zip › data/1.Asymmetry between Vocal Characteristics and Body Size/R studio/Bayesian analysis/analysis results.docx]

**Table 1. Sex differences in acoustic parameters (raw scale and standardized Cohen's d)**

| **Parameter** | **Difference (Hz)** | **95% CrI** | **Cohen's d (median)** | **95% CrI** | **P(women > men)** |
| --- | --- | --- | --- | --- | --- |
| F0 | 97.5 | [77.4, 117.3] | 4.02 | [2.98, 5.06] | 1.000 |
| /a/ Df | 66.0 | [−2.3, 136.3] | 0.75 | [−0.09, 1.56] | 0.971 |
| /u/ Df | 153.8 | [83.7, 225.0] | 1.83 | [0.96, 2.68] | 1.000 |
| /i/ Df | 196.2 | [117.7, 271.9] | 2.07 | [1.18, 2.94] | 1.000 |

*Note.* The difference (Hz) is the posterior mean difference between women and men (women − men), estimated at mean height from the full raw-scale model (parameter ~ height × Sex, weakly informative prior). Cohen's d is derived from the corresponding standardized model (parameter ~ height_z × Sex) as the posterior distribution of the standardized mean difference. P(women > men) is the posterior probability that the raw-scale difference exceeds zero. CrI = 95% credible interval. The CrI for /a/ Df crosses zero, indicating weaker evidence for a sex difference in this parameter.

**Table 2. Within-sex correlations between height and acoustic parameters (raw scale)**

| **Parameter** | **Sex** | **r (median)** | **95% CrI** | **P(r < 0)** |
| --- | --- | --- | --- | --- |
| F0 | Men | −0.10 | [−0.43, 0.22] | 0.732 |
| F0 | Women | −0.21 | [−0.52, 0.10] | 0.908 |
| /a/ Df | Men | −0.27 | [−0.59, 0.05] | 0.956 |
| /a/ Df | Women | −0.33 | [−0.65, −0.02] | 0.982 |
| /u/ Df | Men | −0.27 | [−0.59, 0.04] | 0.957 |
| /u/ Df | Women | −0.03 | [−0.35, 0.30] | 0.562 |
| /i/ Df | Men | −0.23 | [−0.55, 0.09] | 0.921 |
| /i/ Df | Women | −0.20 | [−0.52, 0.12] | 0.893 |

*Note.* Correlation coefficients (r) were derived from within-sex subset regressions (parameter ~ height, raw scale, weakly informative prior) by rescaling the posterior slope: r = β × SD(height) / SD(parameter). With the exception of /a/ Df in women, all 95% credible intervals cross zero, indicating that within-sex associations between height and the acoustic parameters are generally weak and imprecisely estimated.

**Table 3. Within-sex Bayesian regression slopes of acoustic parameters on height (raw scale)**

| **Parameter** | **Sex** | **Slope (mean)** | **95% CrI lower** | **95% CrI upper** | **P(slope < 0)** |
| --- | --- | --- | --- | --- | --- |
| F0 (Hz/cm) | Women | −0.73 | −2.09 | 0.68 | 0.851 |
| F0 (Hz/cm) | Men | −0.94 | −2.18 | 0.32 | 0.933 |
| /a/ Df (Hz/cm) | Women | −5.58 | −9.92 | −1.19 | 0.993 |
| /a/ Df (Hz/cm) | Men | −5.92 | −9.91 | −1.91 | 0.998 |
| /u/ Df (Hz/cm) | Women | −2.44 | −6.75 | 1.90 | 0.867 |
| /u/ Df (Hz/cm) | Men | −3.42 | −7.37 | 0.67 | 0.953 |
| /i/ Df (Hz/cm) | Women | −3.74 | −8.62 | 0.96 | 0.939 |
| /i/ Df (Hz/cm) | Men | −4.88 | −9.26 | −0.53 | 0.986 |

*Note.* Slopes were estimated from within-sex subset models (parameter ~ height, raw scale, weakly informative prior; N = 40 per sex) and are expressed in Hz per centimetre of height. 95% credible intervals exclude zero only for /a/ Df (both sexes) and /i/ Df (men).

**Table 4. Bayesian model comparison for standardized acoustic parameters**

| **Parameter** | **Model** | **R² (median)** | **R² 95% CrI** | **ELPD** | **ΔELPD (vs M1)** | **ΔELPD/SE** |
| --- | --- | --- | --- | --- | --- | --- |
| F0 | M1: height_z | 0.646 | [0.546, 0.704] | −74.70 | — | — |
| F0 | M2: height_z + Sex | 0.846 | [0.809, 0.863] | −43.65 | 31.05 (SE 16.19) | 1.92 |
| F0 | M3: Sex only | 0.844 | [0.807, 0.860] | −43.62 | 31.08 (SE 16.25) | 1.91 |
| /a/ Df | M1: height_z | 0.447 | [0.300, 0.547] | −92.10 | — | — |
| /a/ Df | M2: height_z + Sex | 0.475 | [0.334, 0.570] | −91.60 | 0.50 (SE 8.11) | 0.06 |
| /a/ Df | M3: Sex only | 0.419 | [0.264, 0.527] | −94.59 | −2.49 (SE 9.51) | −0.26 |
| /u/ Df | M1: height_z | 0.483 | [0.347, 0.575] | −89.44 | — | — |
| /u/ Df | M2: height_z + Sex | 0.585 | [0.468, 0.655] | −81.77 | 7.67 (SE 9.09) | 0.84 |
| /u/ Df | M3: Sex only | 0.573 | [0.451, 0.648] | −81.77 | 7.67 (SE 9.06) | 0.85 |
| /i/ Df | M1: height_z | 0.563 | [0.438, 0.639] | −82.83 | — | — |
| /i/ Df | M2: height_z + Sex | 0.673 | [0.579, 0.725] | −72.43 | 10.40 (SE 9.27) | 1.12 |
| /i/ Df | M3: Sex only | 0.659 | [0.564, 0.714] | −73.29 | 9.55 (SE 9.83) | 0.97 |

*Note.* R² is the median and 95% credible interval of the Bayesian R² posterior distribution (computed separately for each model with bayes_R2(fit, summary = FALSE)). Model comparison is based on the expected log predictive density (ELPD) from Pareto-smoothed importance-sampling leave-one-out cross-validation (PSIS-LOO, moment matching); WAIC values are omitted here but were consistent with the LOO-based ranking. ΔELPD/SE values with |ΔELPD/SE| < 1 are treated as weak evidence for a difference between models, following the same threshold used in the prior-sensitivity analysis. Adding Sex to the height-only model produced a credible improvement in fit for F0 (|ΔELPD/SE| ≈ 1.9) but only weak evidence for improvement in the three formant-dispersion measures (/a/, /u/, /i/ Df; |ΔELPD/SE| < 1.2), for which height alone already explains a comparable proportion of variance.

**Table 5. Posterior comparison of height slopes for formant dispersion (Df) versus F0 (standardized scale)**

| **Parameter** | **P(Df slope < F0 slope) — women** | **P(Df slope < F0 slope) — men** |
| --- | --- | --- |
| /a/ Df | 0.866 | 0.913 |
| /u/ Df | 0.236 | 0.906 |
| /i/ Df | 0.554 | 0.826 |

*Note.* Values are posterior probabilities, computed from the standardized full models, that the height slope for the given formant-dispersion (Df) measure is more negative than the height slope for F0, within each sex. In men, this probability is consistently high (0.83–0.91) across all three vowels, suggesting formant dispersion may be more sensitive to height than F0 is. In women, only /a/ Df shows a comparable pattern; evidence is weak for /u/ Df and /i/ Df.
